# Patient and public involvement in mental health research: En route to maturity?

**DOI:** 10.1111/hex.13250

**Published:** 2021-05-20

**Authors:** Alison Faulkner, Mary Chambers

**Affiliations:** ^1^ Independent Service User Researcher London UK; ^2^ Patient and Public Involvement Research Theme Lead for NIHR Applied Research Collaboration South London, a research organisation working to improve health and care services; ^3^ Kingston and St George's Joint Faculty Faculty of Health, Social Care and Education St. George’s University of London London UK

This collection of papers demonstrates a growing interest and maturity of experience within the field of patient and public involvement utilizing a range and various forms of involvement with different groups and communities. Many of the papers placed emphasis on dignity and respect, Faulkner et al, Warner et al.; others, for example Tyler et al., and Kuhne et al., highlighted the importance of listening to the patient voice. The need for shared decision making was referred to by Khan et al., having patients and young people as part of the research team was indicated in the work of Walker et al., where children and young people formed a project advisory group (CYPAG).

The importance of relationships was considered key in the work of Mulvale et al., who incorporated integrative dynamics (ID) approach and experience‐based co‐design (EBCD) to overcome ‘us vs them’ thinking. Photovoice was the method of choice used by Weinstein and colleagues when working with individuals with long‐term mental illness, obesity and living in supportive housing.

There are some careful explorations of appropriate methods to use with different and often marginalized groups and communities; for example, Corvin et al. examined the application of analytic hierarchy process (AHP) to inform the augmentation and implementation of an evidence‐based chronic disease self‐management programme for underserved Latinos living with both minor depression and chronic illness. Thomas and colleagues report an honest reflection of working with people from low‐income backgrounds throughout a research process; Warner et al outline a method of engaging with refugees at the early stages of research; Dewa et al explore the methods and the impact of working co‐productively with young people and saw significant impact on the research, researchers and co‐researchers.

A patient‐targeted feedback intervention after depression screening using the Patient Health Questionnaire (PHQ‐9) was the focus of work by Seeralan et al. It needs to be recognized that for a variety of reasons, patients may not want to participate as outlined by Bixo and colleagues. In their study involving young people being ‘too tired/too sick to participate’ was the most common barrier for non‐participation followed by lack of time and fear of needles. However, the young people identified different ways for increasing the likelihood for participation such as simplification of procedures and information, providing rewards and feedback, and building relationships before asking. The points raised by the young people are equally as applicable to all studies where PPI is a key aspect.

Several papers report on evaluations of the impact of involving service users and/or carers in the research process. In a protocol paper, Littlewood et al describe a longitudinal process of evaluating impact of working with people who self‐harm, the results of which will be interesting to see. Matheson and Weightman take the exploration of process into the therapeutic realm by viewing it as a potential part of the co‐researchers' community re‐integration stage of therapy for complex post‐traumatic stress disorder (CPTSD), contributing to their empowerment through collective action. Morse et al, in working with both service users and carers, identify an ethical dilemma at the heart of the endeavour: the ownership of the story and who can speak for whom. Along with the other studies in this collection, it becomes clear that involvement cannot be regarded as simply instrumental; it has powerful ethical and political threads running through it.

Friesen et al explore some of these ethical dilemmas in more depth, by confronting the limitations of seeking evidence of impact. They suggest that our search for evidence of the impact of involvement obscures the ethical and political grounds behind involvement, the drive to address the imbalance of power within psychiatric knowledge‐making: *'service users have not fought for a voice at the table merely to help improve the research process, but because they have a right to be there'*. They advocate looking beyond impact at the quality of participatory research in relation to the ethical demands of service users, the development of service user capacities and, crucially, sharing power in domains other than just research. This collection of papers suggests a growing maturity in approaches to PPI; however, there is still a need for understanding of the difference between participation, engagement and user‐led research alongside a shared respect for the different forms and fields of knowledge.

